# Show don’t tell: assessing the impact of co-developed patient information videos in paediatric uveitis

**DOI:** 10.1038/s41433-023-02659-w

**Published:** 2023-07-17

**Authors:** Rana Khalil, Salomey Kellett, Harry Petrushkin, Christine Twomey, Jugnoo Rahi, Ameenat Lola Solebo

**Affiliations:** 1 Royal College of Surgeons, Dublin, Ireland; 2 Moorfields Eye Hospital NHS Trust, London, UK; 3 Population, Policy and Practice Programme Department of Research and Teaching, UCL GOS Institute of Child Health, London, UK; 4 Great Ormond Street Hospital for Children NHS Trust, London, UK; 5 Moorfield Eye Hospital Biomedical Research Centre, London, UK

**Keywords:** Uveal diseases, Education

## Abstract

**Background/Objectives:**

There is a paucity of online educational content targeting children and young people with uveitis. We evaluated the impact of a co-designed patient education video on subjective and objective understanding of childhood uveitis.

**Subjects/Methods:**

Co-designed patient education media were produced in collaboration with the Childhood Uveitis Studies steering group and the Great Ormond Street Hospital Generation R Young People’s Advisory Group and narrated by children. Patients managed within the Uveitis service at GOSH were invited to take part in a pre–post survey, undertaken immediately prior to and following viewing of a patient education video.

**Results:**

Forty-three patients participated. These were stratified according to age, duration of disease, and treatment type for analysis. Self-rated knowledge improved across all groups (*p* = 0.001), particularly in those with a new diagnosis of uveitis (*Z* = −8.124, *p* < 0.001). Objective knowledge scores improved across all questions, especially in younger children, those with new disease, and those on steroid only treatment (Z = −3.847, *p* < 0.001, *Z* = −3.975, *p* < 0.001, *Z* = −3.448, *p* < 0.001; respectively). Most participants reported the videos to be easy to understand and with the right amount of information. All stated that they learned something new.

**Conclusions:**

Patient understanding of disease and treatment is crucial to achieving the best possible outcomes for this chronic, relapsing remitting and potentially blinding disorder. Our findings data shows the potential value of co-designed patient information videos, specifically in our study benefitting younger patients and those recently diagnosed. We suggest that other clinical teams could collaborate fruitfully with patient groups to develop similar videos to target possible misinformation and potentially improve patient outcomes.

## Introduction

With the advent of social media and increasing accessibility to the Internet, the use of online media platforms for the access of patient information is at its most prevalent[1]. In 2021, 99% of children were online, with the majority using a mobile phone (72%) or tablet (69%) to do so [2]. Amongst all online platforms, YouTube was by far the most popular [2]. Whilst search engines like Google and YouTube represent a rapidly growing visual library for patients [3], the absence of peer review may result in medical misinformation.

In the last five years, there has been increased interest in the evaluation of the utility of ophthalmology-related YouTube videos, including strabismus, retinitis pigmentosa, cataract surgery, soft contact lenses, refractive surgery, multifocal intraocular lenses, and keratoplasty [4–10]. The conclusion of all these studies was similar: the educational quality of the data presented for patients is inadequate, and reliability poor [4, 11, 12].

Children and young people have become increasingly involved in their own medical decision-making, with particular focus on their ability to assent even if they cannot legally consent to treatment. This is in the context of a commitment by national (e.g., the National Health Service) and supernational (e.g., the United Nations) to ensure participation of children, young people and their families in their experiences of care [13]. As children may not have the same level of health understanding as adults, it has been recommended that “attention should be paid to providing the child with adequate information, as decision-making competence is ‘only as good as the provided information’… this means that the information supplied needs to be adapted to the child’s level of communication and understanding” [14]. The successful use of video based content as an effective way of educating children and young people has been described in multiple clinical settings [15–20]. However, these peer- and consumer- reviewed videos are typically aimed at common conditions or procedures, and are able to draw on the background knowledge of the viewers they are aiming to inform.

Childhood uveitis is a group of chronic, recurring, inflammatory eye diseases, with a collective prevalence of between 4 and 30 per 100,000 [21]. They carry significant risk of both visual loss and negative impact from the potent medications used to prevent visual loss [22]. Multiple systemic therapies may be tried in the early stages of disease (i.e. the first two years). Affected children and their families may also have to deal with other medical problems, such as inflammatory arthritis [21, 22]. Firm patient understanding of disease and treatment is crucial to achieving the good outcomes for this chronic, relapsing remitting and potentially blinding disorder, by supporting concordance and empowering patients as advocates for their care. There is currently a paucity of online educational information targeting children and young people with uveitis. It is unclear whether successful engagement of children, young people and their families could be possible with a video aimed at informing on a rare, complex disorder such as childhood onset uveiti [21].

In this study, we evaluated the impact of a video on childhood uveitis, developed in collaboration with young people affected by the disease, through quantitative evaluation of the video as a patient educational tool, within a quality improvement study [23].

## Materials/subjects and methods

This is a quantitative pre–post study examining the effect of educational videos on self-rated and objective knowledge of uveitis in children and young people.

### Co-development of the video

The video content and format was developed through collaborations with the Great Ormond Street Hospital Generation R Young People’s Advisory Group (GOSH YPAG) [24], and the Childhood Uveitis Studies steering group. This UK disease specific patient advisory group meets three times a year and comprises three young people affected by childhood onset uveitis, including one young person with impaired vision, and three parents of young children with uveitis.

Guidance on the content and images for the videos was gathered via two exercises: (a) through input from the Childhood Uveitis Studies steering group, and (b) through “Show, don’t tell!” posters and suggestion board, designed with support from the GOSH YPAG, and hosted within clinical areas inviting suggestions from the GOSH patient groups. The aim of these consultations was to identify favourite images across the target audience, invite suggestions for alternate images, and determine the key messages about uveitis that these patients would like communicated. Once the storyboard was designed and approved by the Childhood Uveitis Studies steering group, a script was co-produced using words chosen by young people. This was then used to create three, 2-min-long patient and public engagement videos (providing [1] an overview of the disease [2], a description of the different treatments used in uveitis, and [3] an update on uveitis research) narrated by patients aged 8 years to 16 years, their families, and investigators. These were uploaded to YouTube with signposting that the videos sought to inform patients and families on the disease, treatment options and areas of current research. Supplementary material S1 provides a ‘QR’ link to the videos (also available on https://www.youtube.com/watch?v=1vBzqS_HD_E).

### Evaluation of educational quality

Participants in the pre–post study were patients managed within the Uveitis service at Great Ormond Street Hospital. Convenience sampling was used, with families invited to take part on attendance to clinic appointments, but before the consultation. Patients (and their families) who had previously seen or been involved in the development of the video were excluded from the invitation to participate. The pre–post impact survey was undertaken immediately prior to and following watching of the first video (on disease) on YouTube. An impact survey (Supplementary S2) was developed de novo under the guidance of the Childhood Uveitis Studies steering group, and informed by previous work by other paediatric patient and public involvement and engagement groups [20]. The survey was hosted online and completed anonymously whilst the patient was in clinic (with the investigator out of view of the participants screen) on a study laptop. The first three questions enquired about participant characteristics (age of disease onset, current age, and which treatments or complications the patients had experienced). Before watching the video, participants were asked three questions involving self-rated uveitis knowledge, then six questions testing objective knowledge. After the video, two self-rated questions were repeated followed by six objective knowledge questions (Supplementary S2). Four satisfaction questions were also included at the end. Children aged 12 years and over were asked to complete the survey themselves, and younger children completed the survey with help from their parents / carers. Participants were approached sequentially on attendance at the Uveitis clinic at Great Ormond Street.

### Analysis

The SPSS software, Version 29.0 (SPSS Inc., Chicago, IL, USA), was used for statistical analysis. A descriptive analysis was undertaken of patient characteristics. The degree of positive or negative changes in both self-reported and objective knowledge of disease were chosen as the target outcomes for this patient educational tool, fulfilling the ‘SMART’ quality improvement criteria (Specific (S), Measurable (M), Achievable (A), Realistic (R), and Timely (T)) [23]. Differences between the timepoint before watching the video (T1) and the timepoint after watching the video (T2) were measured using nonparametric statistics (Wilcoxon Signed Ranks Test). Sub group analysis was undertaken using the following groupings: age at watching the video <12 years versus ≥12 years, newly diagnosed (<2 years) versus established (≥2 years) disease, treatment with steroids versus disease modifying agents (such as methotrexate) versus biologics (such as adalimumab), and complications (specifically glaucoma, cataract and macular oedema) versus no complications. Since our three subgroups were not normally distributed, the Mann–Whitney *U* test was used to compare outcome variables based on differences in age or duration of disease. The Friedman test was used to evaluate whether the differences between the three treatment subgroups had an impact on disease knowledge outcomes. The Friedman Test was also used to assess the impact of patient characteristics (age, duration of disease, type of treatment) on outcomes. All tests are two-sided and a *p* value < 0.05 was considered significant.

The conduct of this work was adherent to the tenets of the Declaration of Helsinki and the study received the necessary institutional approvals for a quality improvement project.

## Results

### Participant characteristics

The survey was administered to 43 children, young people and families (of the 64 approached) within the paediatric uveitis service.

Participant characteristics are shown in Table 1. The mean age was 10.9 years (SD 3.3, range 2–16, median 11), with 51% of participants under the age of 12 years. The average duration of disease was 3.2 years (SD 3.5, range 0-13, median 2), with 49% of patients reporting a duration of under 2 years. In terms of treatment, the majority had received steroid eye drops or steroid tablets plus at least one other immunomodulatory agent.

**Table 1:** Participant characteristics (*n* = 43).

Characteristic	% (*n*)
Age, years	
Mean (SD)	10.9 (3.3)
<12	51% (22)
≥12	49% (21)
Duration of disease, years	
Mean (SD)	3.2 (3.5)
<2	49% (21)
≥2	51% (22)
Treatment	
Steroid drops or tablets only	35% (15)
Steroids drops or tablets (and) at least one other agent	44% (19)
Steroid drops or tablets (and) at least one other agent (and) at least one surgical intervention	21% (9)

### Self-rated knowledge

Self-rated knowledge scores were compared using the Wilcoxon Signed Ranks Test (Table 2). Answers of ‘not sure’ were classified as absence of knowledge. Self-marked understanding of uveitis improved significantly after watching the video (*p* = 0.001). Self-marked knowledge of all the tested terms (‘uvea’, ‘inflammation’, ‘glaucoma’, ‘cataract’, and ‘macular oedema’) significantly improved after video viewing (*p* < 0.001), with the most benefit seen for the words ‘uvea’ and ‘macular oedema’. Although, those with a new diagnosis of uveitis (<2 years) had a greater degree of improvement in subjective knowledge compared to those with established disease (*Z* = −8.124, *p* < 0.001 and *Z* = −5.745, *p* < 0.001; respectively).

**Table 2:** Subjective knowledge (*n* = 43).

	% (*n*)	% (*n*)		
Measure	Pre	Post	Test statistic, *P* value	
How would you describe your understanding of uveitis?				
None	19% (8)	0% (0)	*Z* = −3.258	
Some	49% (21)	49% (21)	***p*** = **0.001**	
Good	33% (14)	51% (22)		
Heard of the word uvea?				
Yes	91% (39)	N/A		
No	9% (4)	N/A		
Unsure	0% (0)	N/A		
Heard of the word inflammation?				
Yes	100% (43)	N/A		
No	0% (0)	N/A		
Unsure	0% (0)	N/A		
Heard of the word glaucoma?				
Yes	72% (31)	N/A		
No	28% (12)	N/A		
Unsure	0% (0)	N/A		
Heard of the word cataract?				
Yes	86% (37)	N/A		
No	14% (6)	N/A		
Unsure	0% (0)	N/A		
Heard of the words macular oedema?				
Yes	23% (10)	N/A		
No	30% (13)	N/A		
Unsure	47% (20)	N/A		
Know what uvea means?				
Yes	21% (9)	100% (43)	*Z* = −5.831	
No	7% (3)	0% (0)	***p*** = **<0.001**	
Unsure	72% (31)	0% (0)		
Know what inflammation means?				
Yes	63% (27)	100% (43)	Z = −4.000	
No	0% (0)	0% (0)	***p*** < **0.001**	
Unsure	37% (16)	0% (0)		
Know what glaucoma means?				
Yes	70% (30)	100% (43)	Z = −3.606	
No	14% (6)	0% (0)	**p** < **0.001**	
Unsure	16% (7)	0% (0)		
Know what cataract means?				
Yes	70% (30)	100% (43)	Z = −3.606	
No	14% (6)	0% (0)	***p*** < **0.001**	
Unsure	16% (7)	0% (0)		
Know what macular oedema means?				
Yes	23% (10)	95% (41)	Z = −5.568	
No	30% (13)	2% (1)	***p*** < **0.001**	
Unsure	47% (20)	2% (1)		

In some cases, rounding up to zero decimals does not equal to a total of 100%. Subjective knowledge scores were compared using the Wilcoxon Signed Ranks test. Note: *p* values in bold indicate significance less than 0.05.

### Objective knowledge

Objective knowledge scores were also compared using the Wilcoxon Signed Ranks test (Table 3). Answers of ‘don’t know’ were classified as absence of knowledge, as were incorrect answers. There was a statistically significant improvement in scores across all six questions, with a particular increase in the correct identification as false of statements 2 (that ‘Inflammation means that part of the eye has an infection’; *Z* = −5.292, *p* < 0.001), 3 (‘Once you have uveitis it never goes away’; *Z* = −5.477, *p* < 0.001), and 5 (‘Cataract is when scar tissue crosses the front of the eye; *Z* = −5.477, *p* < 0.001).

**Table. 3:** Objective knowledge overall.

	% Correct answer (*n*)	% Correct answer (*n*)		
Measure	Pre	Post	Test statistic, *P* value	
Question 1: The iris is part of the uvea (true)	56% (24)	100% (43)	*Z* = −4.359, ***p*** < **0.001**	
Question 2: Inflammation means that part of the eye has an infection (false)	35% (15)	100% (43)	*Z* = −5.292, ***p*** < **0.001**	
Question 3: Once you have uveitis it never goes away (false)	21% (9)	91% (39)	*Z* = −5.477, ***p*** < **0.001**	
Question 4: Children with uveitis can have high eye pressures (true)	77% (33)	100% (43)	*Z* = −3.162, ***p*** = **0.002**	
Question 5: Cataract is when scar tissue crosses the front of the eye (false)	21% (9)	91% (39)	*Z* = −5.477, ***p*** < **0.001**	
Question 6: Biologic agents are more targeted for uveitis than traditional treatments like methotrexate (true)	60% (26)	98% (42)	*Z* = −3.771, ***p*** < **0.001**	

Overall objective knowledge scores were compared using the Wilcoxon Signed Ranks test. Note: *p* values in bold indicate significance less than 0.05.

To investigate whether age (under 12 years versus 12 years or older) or duration of disease (new, i.e., under 2 years, versus established disease of 2 years or longer duration) had an impact on improvement in objective knowledge (total possible score of 6; 1 for each question), the Mann–Whitney *U* test was used. Both variables were found to have a statistically significant impact on outcomes (*Z* = −1.991, *p* = 0.047 and *Z* = −2.469, *p* = 0.014; respectively). There was a mean 3.73 net marks (SD 2.004) improvement for participants under 12 years, compared to a mean 2.52 net marks (SD 1.914) for those 12 years or older. Those with duration of disease under 2 years had a mean net improvement of 3.90 marks (SD 1.58), whilst the rest had a mean net improvement of 2.3 marks (SD 2.03).

To test the effect of treatment history on net objective scores, the Friedman Test was used. A score was assigned (out of a possible total of 6) for answers at T1 and T2. Then, the difference between both scores was calculated to determine the net improvement in objective knowledge. The biggest improvement was seen in the first treatment group (steroid drops or tablets only, mean 4.44, SD 1.424), with the second (steroids and at least one other agent, mean 2.78, SD 2.279) and third (steroids and at least one other agent and at least one surgical intervention, mean 1.89, SD 1.965) following in respective order. There was a statistically significant difference in net improvement of objective knowledge depending on which treatment group each participant belonged to, *χ*^2^ [2] = 11.455, *p* = 0.003. Post hoc analysis with Wilcoxon signed-rank tests was conducted with a Bonferroni correction applied, resulting in a significance level set at *p* < 0.017. Median (IQR) net improvement in objective knowledge for treatment group 1, group 2 and group 3 were 5 (3–5), 3 (0–5), and 2 (0–3.5), respectively.

There were no significant differences in knowledge improvement between groups 1 and 2 (Z = -1.209, *p* = 0.227), or between groups 2 and 3 (*Z* = −0.851, *p* = 0.395). However, there was a statistically significant difference between groups 1 and 3 (*Z* = −2.694, *p* = 0.007). To compare the impact of various patient characteristics on outcome variables, the Wilcoxon Signed Ranks test was again used. There was a statistically significant difference noted for all demographics, with the most significant for those with disease duration under 2 years (*Z* = −3.975, *p* < 0.001) (Table 4).

**Table 4:** Impact of patient characteristics on objective outcome variables.

Characteristic	Test statistic, *P* value
Age <12 years	Z = −3.847, ***p*** < **0.001**
Age ≥12 years	Z = −3.471, ***p*** < **0.001**
Duration of disease < 2 years	Z = −3.975, ***p*** < **0.001**
Duration of disease ≥ 2 years	Z = −.214, ***p*** = **0.027**
Treatment group 1 (steroid drops or tablets only)	Z = −3.448, ***p*** < **0.001**
Treatment group 2 (steroids and at least one other agent)	Z = −2.433, ***p*** = **0.015**
Treatment group 3 (steroids and at least one other agent and at least one surgical intervention)	Z = −2.032, ***p*** = **0.042**

Objective knowledge scores were compared using the Wilcoxon Signed Ranks test. Note: *p* values in bold indicate significance less than 0.05.

### Change score

**Fig. 1:**
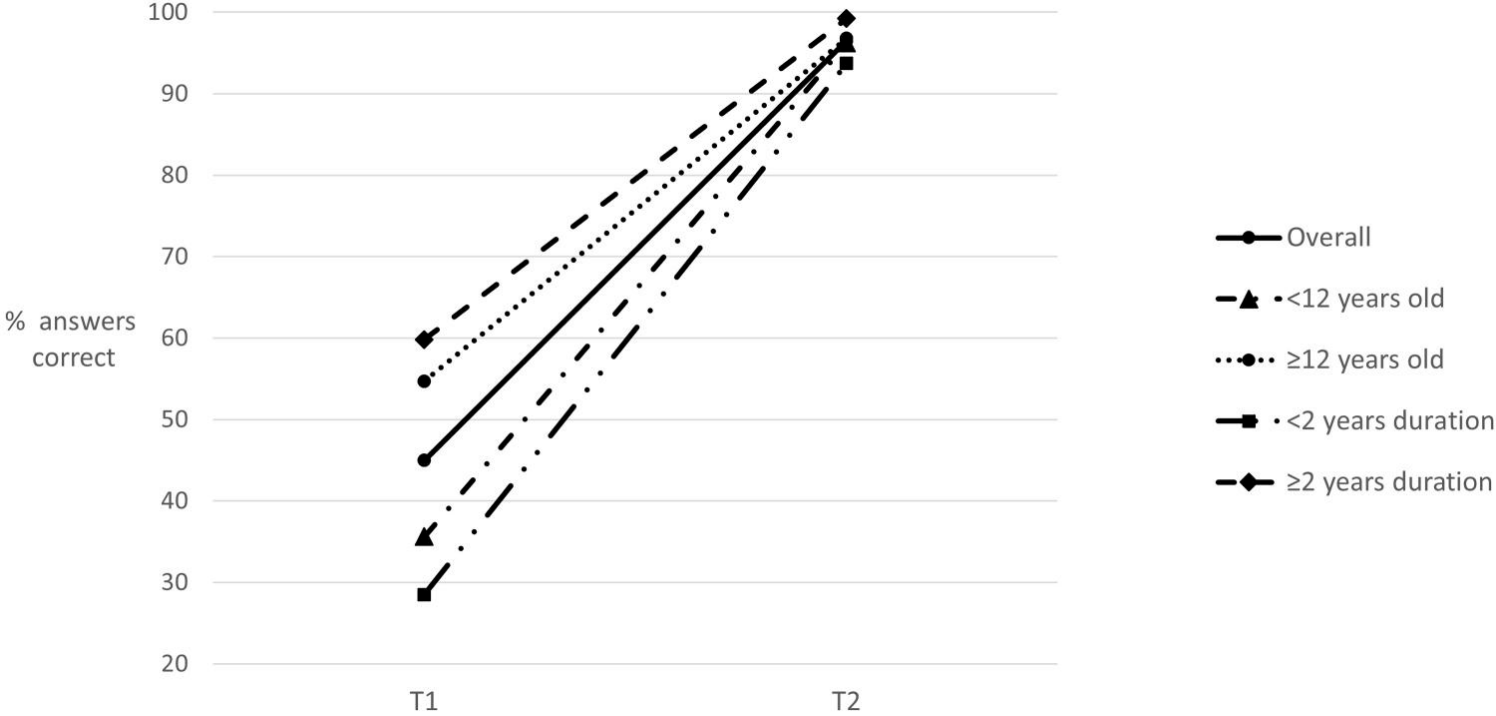
Changes in understanding overall and within patient age and disease duration subgroups. Disease knowledge pre (T1) and post (T2) video intervention.

There was an overall increase in objective knowledge across all groups (Fig. 1). The T1–T2 change score was greater for those aged less than 12 years compared to older children (mean rank 25.64 vs 18.19 respectively, *U* = 151, *Z* = −1.991, *p* = 0.047), as well as for those with less than 2 years of disease compared to longer (mean rank 29.36 vs 14.98 respectively, *U* = 76.50, *Z* = −3.917, *p* < 0.001).

There was no statistically significant difference in T1–T2 change score between treatment groups 1 and 2 or groups 2 and 3. However, there was a significant improvement when comparing treatment groups 1–3 (mean rank 15.2 vs 8 respectively, *U* = 27, *Z* = −2.485, *p* = 0.13).

### Satisfaction

Overall, there was a good level of satisfaction with the videos, with 65% of participants stating they learned something new (Supplementary Table, S3). All participants rated the videos as very easy or quite easy to understand, with 77% and 70% stating that the amount of information and length of videos were just right, respectively.

A common theme encountered in the free text section at the end of the survey was the need for more information (8 of 43 families); this was across varying patient groups. There was particularly good feedback regarding the use of children’s voices, and agreement that these videos would be helpful for new families. One parent of an 11-year-old with an established diagnosis wrote “*Would have liked this video when my son was younger - and was good for him to watch*”, while one 14-year-old commented “*Good video for new families*”.

## Discussion

From this quantitative pre–post study we report a positive impact of a co-developed patient educational video on the self-reported and objective knowledge of childhood uveitis in children, young people and families affected by the disease. This positive impact was seen irrespective of disease duration, age of the affected child, or disease severity (as measured using level of treatment given or presence of disease-related complications).

Our results show that our co-developed video content subjectively and objectively improved participants’ understanding of paediatric uveitis. The most benefit in subjective knowledge was seen for the words ‘uvea’ and ‘macular oedema’. Age <12 years old and disease duration <2 years had the greatest impact on outcome variables. This suggests that younger patients and those with newly diagnosed disease (particularly in the first year) benefit the most from targeted educational content. The most significant difference within the treatment groups was seen between groups 1 and 3, supporting the theory that patients on the lowest treatment regimen for uveitis benefit the most from educational content compared to those who have had complications and have had the lived experience of complex disease.

Whilst not directly comparable to ours, a recent study assessing the quality of uveitis-related YouTube videos identified a large viewership for this condition, with some content attracting more than 30,000 views per year [11], The authors reported that viewer satisfaction, or the video power index (VPI, a metric derived from the ratio of the number of viewer ‘likes’ to the number of views) was highest for patient education videos (when compared to patient experience or medical education videos) [11]. However, the mean Global Quality Score (a 5 point scale, from 1 to a best possible score of 5) of the Uveitis patient education video was 2.8 (below medium quality). Other investigators have suggested that YouTube users may prefer videos of ‘low quality’, which are often simpler and avoid technical terms [4]. Videos published by academic institutions were found to have high quality scores but low VPI scores, due to a lack of signposting regarding the target audience, with content aimed at medical professionals, and therefore above the level of understanding of most patients [8, 11]. In terms of optimum video duration so as not to lose viewership, whilst the most popular videos on YouTube are less than ten minutes long [12], the average uveitis video duration was at least 20 min [11].

Our videos were well received in terms of content, length, and ease of understanding. Whilst ongoing patient education is important, we suggest that the most impactful time (with regards to disease trajectory and later life outcomes) at which to educate patients for any chronic disease is early in the disease course. Thus, it is particularly encouraging that the greatest impact was seen for those with shorter disease durations, and we recommend that families are directed towards the video soon after diagnosis. Although those with longer duration of disease and increased severity do appear to benefit less, implying that existing educational materials have already been of some benefit in this group, it is notable that more than a third of respondents with longer established disease still demonstrated limitations in disease understanding. The video content will also be of benefit to those with established disease and may be particularly useful for informing those children aged 12 years and over who were diagnosed early in life, and who are undergoing the transition to greater self-care and empowerment. As age at onset of childhood uveitis follows a bimodal pattern, with the first peak at age 2–5 years, these young people may have not been fully informed during the education of the family on disease and treatment at the initial milestone of diagnosis.

### Strengths and limitations

This study employed a ‘patients as partners’ approach, from engineering the ideas for intervention to designing the educational content. This takes into account the wishes and preferences of children and young people in their treatment plans, whilst understanding that their communication needs may be different to adults [13]. The involvement of a patient expert group rather than, for example, the use of an interview with one or two patients, enabled a diversity of experiences to inform the video, supporting wider engagement across a patient population. The co-production of the storyboard and script, as well as narration by children is a major strength of this study, as it renders the content relatable to the target audience. The use of YouTube videos in information delivery represents an up-to-date and user friendly feature, while the involvement of relevant stakeholders including current patients in the GOSH service and specific patient-led groups reiterates the need for co-design in healthcare interventions [25, 26]. The quantitative assessment in this study was performed in a real clinical setting with patients affected by disease, each of whom had various degrees of pre-existing uveitis-related knowledge. This provides more realistic and broadly applicable results.

A limitation of the study was the relatively small sample population, however, this is in the context of rare disease. It was therefore not possible to undertake multivariate analysis of outcomes. Certainly, all subgroups showed positive outcomes to the videos, however it is difficult to know if some truly benefited over others. A larger sample size in future might mitigate this. Whilst the lived experience of uveitis informed these videos, the pre–post study sample did not involve any families with visual impairment (i.e., poor vision with both eyes open). The accessibility of these videos to those with sensory impairments is unclear, although the involvement of families in script development should support video utility amongst families affected by visual disability. Additionally, this study was limited to English-speaking participants only. In future, translations into the most common languages encountered at GOSH (and indeed, across the UK) would ensure greater patient inclusion and reduce health information disparities due to language barriers. This study is also only able to report on short term recall of video contents, and it is possible that long term retention is poor. However, hosting of this video on a publicly available site allows patients and families to refresh knowledge as needed.

In conclusion, we developed a co-designed patient education resource targeted at children, young people and their families with paediatric uveitis. This is online, easily accessible, and widely applicable to patient populations across the UK, and in other English-speaking countries. The video can also be translated for use in other populations. Regular review of the video may be necessary to support adaptability and sustainability of content, for example through the future use of supplementary audio files or podcasts. There is a need for further high quality, relevant, and reliable online patient information for paediatric onset eye disorders.

## Summary

### What was known before

- Childhood uveitis is a group of rare, chronic, recurring, inflammatory eye diseases.
- Firm patient understanding of disease and treatment is crucial to achieving good outcomes for this relapsing remitting and potentially blinding disorder.
- There is a paucity of online educational content targeting children and young people with uveitis.
- Whilst search engines like Google and YouTube represent a rapidly growing visual library for patients, the absence of peer review can result in biased information and subsequent ill-informed decisions.

### What this study adds

- From this quantitative pre–post study we report a positive impact of a co-developed patient educational video on the self-reported and objective knowledge of childhood uveitis in children, young people and families affected by the disease.
- This positive impact was seen irrespective of disease duration, age of the affected child, or disease severity (as measured using level of treatment given or presence of disease-related complications).
- The co-production of the storyboard and script, peer review of educational content by the authors, as well as narration by children is a major strength of this study, as it renders the content relatable to the target audience.

### Supplementary information

Supplementary data S1

Supplementary data S2

Supplementary data 3

EYE checklist

## Data Availability

The datasets generated during and/or analysed during the current study are available from the corresponding author on reasonable request.
