# Supplementary figures and images for: Show don’t tell: assessing the impact of co-developed patient information videos in paediatric uveitis
Source: Eye (Lond). 2023 Jul 17;38(2):246–52. doi: 10.1038/s41433-023-02659-w (PMC10810776; doi:10.1038/s41433-023-02659-w)

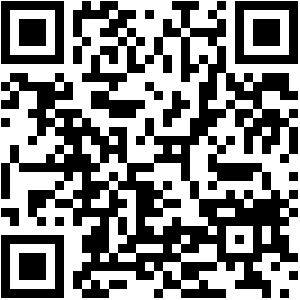

Supplement: Supplementary file 1 — Supplementary data S1 [file 41433_2023_2659_MOESM1_ESM.jpg]
