# Supplementary material for: Show don’t tell: assessing the impact of co-developed patient information videos in paediatric uveitis
Source: Eye (Lond). 2023 Jul 17;38(2):246–52. doi: 10.1038/s41433-023-02659-w (PMC10810776; doi:10.1038/s41433-023-02659-w)
Supplement: Supplementary file 2 — Supplementary data S2 [file 41433_2023_2659_MOESM2_ESM.docx]

Supplementary S2: Surveys

**Some questions about you / your child with uveitis:**

1. How old are you?..............

2. How old were you when the uveitis started?............

3. Which treatments have you had for uveitis? (mark all that apply)

| Steroid drops | Steroid tablets |  |
| --- | --- | --- |
| Methotrexate | Mycophenolate Mofetil | Adalimumab |
| Another biologic | Another non-biologic |  |
| Cataract surgery | Glaucoma surgery |  |

**QUESTIONNAIRE 1**

**Some questions about your understanding of uveitis**

4. How would you describe your understanding of uveitis?

| None | Some | Good |
| --- | --- | --- |

5. Have you heard of these words or terms before?

| *Uvea* | yes | no |
| --- | --- | --- |
| *Inflammation* | yes | no |
| *Glaucoma* | yes | no |
| *Cataract* | yes | no |
| *Macular oedema* | yes | no |

6. Do you know what these terms mean?

| *Uvea* | yes | no | not sure |
| --- | --- | --- | --- |
| *Inflammation* | yes | no | not sure |
| *Glaucoma* | yes | no | not sure |
| *Cataract* | yes | no | not sure |
| *Macular oedema* | yes | no | not sure |

**Read the following statements and for each one answer True, False or Don’t know**

|  | True | False | Don’t know |
| --- | --- | --- | --- |
| 7. The iris is part of the uvea |  |  |  |
| 8. Inflammation means that part of the eye has an infection |  |  |  |
| 9. Once you have uveitis it never goes away |  |  |  |
| 10. Children with uveitis can have high eye pressures |  |  |  |
| 11. Cataract is when scar tissue crosses the front of the eye |  |  |  |
| 12. Biologic agents are more targeted for uveitis than traditional treatments like methotrexate |  |  |  |

**QUESTIONNAIRE 2**

**Some questions about the video**

**1. Were the videos easy or hard to understand?**

| Very easy | Quite easy | Quite hard | Very hard |
| --- | --- | --- | --- |

**2. The amount of information in the videos was:**

| Too much | Too little | The right amount |
| --- | --- | --- |

**3. The length of the videos were:**

| Too long | Too short | The right amount |
| --- | --- | --- |

**4. Did you learn anything new?**

| Yes | No | Not sure |
| --- | --- | --- |

**Some questions about your understanding**

5. How would you describe your understanding of uveitis?

| None | Some | Good |
| --- | --- | --- |

6. Do you know what these terms mean?

| *Uvea* | yes | no | not sure |
| --- | --- | --- | --- |
| *Inflammation* | yes | no | not sure |
| *Glaucoma* | yes | no | not sure |
| *Cataract* | yes | no | not sure |
| *Macular oedema* | yes | no | not sure |

**Read the following statements and for each one answer True, False or Don’t know**

|  | True | False | Don’t know |
| --- | --- | --- | --- |
| 7. The iris is part of the uvea |  |  |  |
| 8. Inflammation means that part of the eye has an infection |  |  |  |
| 9. Once you have uveitis it never goes away |  |  |  |
| 10. Children with uveitis can have high eye pressures |  |  |  |
| 11. Cataract is when scar tissue crosses the front of the eye |  |  |  |
| 12. Biologic agents are more targeted for uveitis than traditional treatments like methotrexate |  |  |  |

**Finally, in your own words, let us know what you thought of the animation and if there is any other information you would have liked in it:**
