# Supplementary material for: Show don’t tell: assessing the impact of co-developed patient information videos in paediatric uveitis
Source: Eye (Lond). 2023 Jul 17;38(2):246–52. doi: 10.1038/s41433-023-02659-w (PMC10810776; doi:10.1038/s41433-023-02659-w)
Supplement: Supplementary file 3 — Supplementary data 3 [file 41433_2023_2659_MOESM3_ESM.docx]

| Supplementary table (S3): Video satisfaction | | |
| --- | --- | --- |
|  |  |  |
| Measure |  | % (n) |
| Were the videos easy or hard to understand? | | |
| Very easy |  | 19% (8) |
| Quite easy | | 81% (35) |
| Very hard |  | 0% (0) |
| Quite hard | | 0% (0) |
| The amount of information in the videos was: | | |
| Too much | | 0% (0) |
| Too little |  | 23% (10) |
| The right amount | | 77% (33) |
| The length of the videos were: | | |
| Too long |  | 0% (0) |
| Too short |  | 30% (13) |
| The right amount | | 70% (30) |
| Did you learn anything new? | | |
| Yes |  | 65% (28) |
| No |  | 0% (0) |
| Not sure |  | 35% (15) |
